# SLC44A2-mediated phenotypic switch of vascular smooth muscle cells contributes to aortic aneurysm

**DOI:** 10.1172/JCI183527

**Published:** 2024-08-15

**Authors:** Mengen Xing, Wanqi Chen, Yachen Ji, Weihong Song

**Affiliations:** 1 Institute of Aging, Key Laboratory of Alzheimer’s Disease of Zhejiang Province, School of Mental Health and Affiliated Kangning Hospital, The First Affiliated Hospital, Wenzhou Medical University, Wenzhou, Zhejiang, China.; 2 Oujiang Laboratory (Zhejiang Lab for Regenerative Medicine, Vision and Brain Health), Wenzhou, Zhejiang, China.

**Keywords:** Vascular biology, Molecular biology

## Abstract

The phenotypic switch of vascular smooth cells (VSMCs) from a contractile to a synthetic state is associated with the development and progression of aortic aneurysm (AA). However, the mechanism underlying this process remains unclear. In this issue of the *JCI*, Song et al. identified SLC44A2 as a regulator of the phenotypic switch in VSMCs. Inhibition of SLC44A2 facilitated the switch to the synthetic state, contributing to the development of AA. Mechanistically, SLC44A2 interacted with NRP1 and ITGB3 to activate the TGF-β/SMAD signaling pathway, resulting in VSMCs with a contractile phenotype. Furthermore, VSMC-specific SLC44A2 overexpression by genetic or pharmacological manipulation reduced AA in mouse models. These findings suggest the potential of targeting the SLC44A2 signaling pathway for AA prevention and treatment.

## Aortic aneurysm and the phenotypic switch of VSMCs

Aortic aneurysm (AA) is characterized by a permanently localized enlargement or expansion of the aorta, involving vascular inflammation and damage to the extracellular matrix (ECM) (1). AA is recognized as the ninth-leading cause of death overall and the second-most prevalent aortic disease after atherosclerosis (2), with its incidence at approximately 2.8 per 100,000 individuals (3). The rupture of an AA is life-threatening, often leading to hemorrhage-mediated deaths (2). However, our understanding of risk factors and molecular mechanisms underlying AA development and progression remains limited.

The development of AA is a complicated process involving multiple cell types (4), with vascular smooth muscle cells (VSMCs) being among the most influential. VSMCs are crucial regulators of ECM net composition and play a critical role in preserving vasoconstriction and vasodilatation. The phenotypic switch of VSMCs, which involves changing from a contractile state into a synthetic state (5), is accompanied by ECM damage and vascular inflammation under pathologic conditions, and has been demonstrated to be crucial in the development and progression of AA (4). During this process, excessive matrix metalloproteinases (MMPs) are secreted by proinflammatory cells (4), leading to ECM remodeling accompanied by abnormal activation of transforming growth factor β (TGF-β) (6). However, the pathogenic mechanism underlying the VSMC phenotypic switch in AA remains poorly defined.

In this issue of the *JCI*, Song et al. (7) initially analyzed the data from two published data sets, VSMC phenotype–related genes (8) and differentially expressed markers for VSMCs (9), combined with two databases from Human Aortic Aneurysm (GSE47472) and Mouse Aortic Aneurysm (GSE186865), and identified *SLC44A2* as a key gene associated with AA with highest confidence for further analysis (7).

## The suppressive effect of SLC44A2 in AA

*SLC44A2*, also known as *CTL2* (choline transporter–like protein 2), is a gene located on chromosome 19p13.2 with 24 exons. SLC44A2 plays a crucial role in choline and ethanolamine transport and uptake (10, 11). SLC44A2 is expressed in variety of human tissues, including the inner ear, lung, kidney, and blood cells (10), and is located in the mitochondrion membrane and cell membrane. Genome-wide association studies (GWAS) have identified SLC44A2 as a critical factor closely linked with several human phenotypes, such as hearing loss, lung jury, Ménière disease, and venous thrombosis (12, 13). However, its role in cardiovascular disease remains unknown.

**Figure 1:**
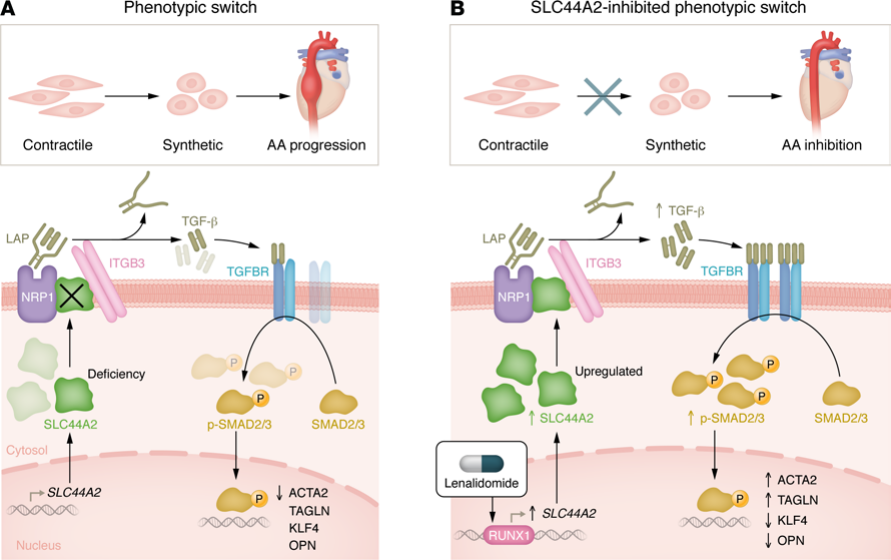
SLC44A2 mediates the VSMC phenotypic switch in aortic aneurysm. (**A**) The switch from a contractile to a synthetic state is crucial for the development and progression of aortic aneurysm. SLC44A2 functions as a scaffolding protein that regulates the TGF-β/SMAD signaling pathway by forming a complex with NRP1 and ITGB3. This complex facilitates the release of activated TGF-β from latent TGF-β (latency-associated peptide [LAP]), which then binds to TGF-β receptors (TGFBRs) on the cell membrane, initiating the downstream phosphorylation of SMAD2/3. p-SMAD2/3 then translocates into the nucleus, where it regulates the expression of contractile markers ACTA2 and TAGLN, or synthetic markers KLF4 and OPN, associated with the VSMC phenotypic switch. SLC44A2 deficiency induced by siSLC44A2 disrupts the association between NRP1 and ITGB3, reduces the levels of TGF-β and p-SMAD2, and promotes the expression of synthetic VSMC markers KLF4 and OPN, while suppressing contractile VSMC markers ACTA2 and TAGLN, further contributing to the pathogenesis of aortic aneurysm. (**B**) RUNX1 acts as a key regulator of SLC44A2 by binding to the *SLC44A2* promoter region and transcriptionally activating *SLC44A2* gene expression. Lenalidomide (LEN), an activator of RUNX1, enhances SLC44A2 expression, boosting the SLC44A2/NRP1/ITGB3/TGF-β/SMAD signaling pathway. This promotes the expression of VSMC contractile markers ACTA2 and TAGLN, and represses synthetic VSMC markers KLF4 and OPN, inhibiting the phenotypic switching of VSMCs from a contractile to a synthetic state, ultimately suppressing the development of aortic aneurysm.

Song and colleagues (7) provide compelling evidence that SLC44A2-mediated VSMC phenotypic switching plays an essential role in the pathogenesis of AA (Figure 1). The authors initially observed changes in SLC44A2 expression between primary mouse aortic smooth muscle cells (MASMCs) from saline-infused mice and those from angiotensin II–infused (Ang II–infused) mice. Analysis of single-cell RNA sequencing (scRNA-seq) data demonstrated predominant SLC44A2 expression in VSMCs. Notably, both Ang II–infused mice and human aortic smooth muscle cells (HASMCs) displayed increased SLC44A2 expression. Furthermore, SLC44A2 levels were upregulated in human patients with abdominal AA (AAA), indicating a crucial role for SLC44A2 in AA development. The authors further investigated the regulatory role of SLC44A2 in the VSMC phenotypic switch by silencing or overexpressing SLC44A2 in HASMCs or MASMCs. Knockdown of SLC44A2 suppressed HASMC or MASMC contractility, whereas overexpression of SLC44A2 enhanced VSMC contractile markers (ACTA2 and TAGLN) and repressed synthetic markers (OPN and KLF4), along with reduced MMP activity. Importantly, reintroduction of SLC44A2 into SLC44A2-deficient HASMCs or MASMCs inhibited the phenotypic switch of VSMCs from a contractile to synthetic state. These data indicate that SLC44A2 is of vital importance in preserving VSMCs’ contractile phenotype.

Song and authors further examined the role of SLC44A2 in AA progression using *Apoe^–/–^* *Tagln*^Cre/+^ mice. Overexpression of SLC44A2 by injecting lentivirus carrying an *Slc44a2* overexpression plasmid (Lenti-*Slc44a2*) ameliorated Ang II–induced elastin damage, medial degeneration, and arterial dilatation, suggesting a protective role of SLC44A2 in AA. Conversely, SLC44A2 deficiency in mice (*Slc44a2*^SMKO^ mice) aggregated the VSMC phenotypic switch to the synthetic state and exacerbated AA progression. Collectively, these data demonstrated that SLC44A2-mediated regulation of the VSMC phenotype is critical in AA progression, highlighting its potential as a therapeutic target in managing AAs (7).

## The SLC44A2/NRP1/ITGB3 axis preserves the VSMC contractile phenotype

To elucidate the mechanism underlying SLC44A2-regulated downstream signaling pathways involved in VSMC phenotypic switching and AA development, Song et al. (7) employed a combined coimmunoprecipitation assay with mass spectrometry scanning and demonstrated an interaction between SLC44A2 and neuropilin 1 (NRP1), which is associated with TGF-β signaling (Figure 1). This interaction was further confirmed in HASMCs, and it was found to be strengthened in *Apoe^–/–^* and Lenti-*Slc44a2* mice under Ang II induction, indicating the critical role of the SLC44A2-NRP1 interaction in AA. Overexpression of SLC44A2 elevated the levels of TGF-β and resulted in phosphorylated SMAD2/3 (p-SMAD2/3). The activation of TGF-β signaling by SLC44A2 is dependent on NRP1, as NRP1 knockdown inhibited SLC44A2-mediated increases in TGF-β, p-SMAD2/3, and the contractile phenotype of VSMCs under Ang II stimulation.

Further mechanistic studies by Song et al. (7) demonstrated that deletion of the MAM domain in NRP1 inhibited the SLC44A2-NRP1 interaction by cotransfecting different NRP1-mutant plasmids with an SLC44A2 plasmid. Meanwhile, deletion of residues 505–569 in SLC44A2 inhibited its interaction with NRP1 and reduced the downstream TGF-β signaling. Moreover, knockout of residues 55–232 in SLC44A2 disrupted TGF-β signaling and the binding between SLC44A2 and ITGB3. Furthermore, SLC44A2 ablation caused a disassociation between NRP1 and ITGB3. Interestingly, knockdown of ITGB3 decreased the SLC44A2-upregulated TGF-β, p-SMAD2/3, and contractile phenotypes when induced by Ang II. Remarkably, Song et al. (7) provided evidence showing the regulatory effect of the SLC44A2/NRP1/ITGB3 axis on TGF-β activation in AA progression (Figure 1). However, further confirmation in NRP1-knockout mice is warranted to support this exciting discovery. Follow-up experiments will further enhance our understanding of SLC44A2 modulations in AA.

## The therapeutic potential of lenalidomide for AA

In the Song et al. report (7), the authors further identified Runt-related transcription factor 1 (RUNX1) as a regulator to transcriptionally activate *SLC44A2* gene expression. RUNX1 upregulates *SLC44A2* gene expression by enhancing its promoter activity in MASMCs and HASMCs. Upregulation of RUNX1 is accountable for the induction of SLC44A2 during AA as a compensatory mechanism. Song et al. (7) demonstrated that lenalidomide (LEN), an upregulator of RUNX1 in hematopoietic stem and progenitor cells (14), was capable of stimulating the expression of both RUNX1 and SLC44A2. Importantly, LEN administration in mice rescued AA with no side effects, alleviating VSMC dedifferentiation in aortic tissues. This result was evident by increased ACTA2 and decreased KLF4 levels, along with higher levels of TGF-β and p-SMAD2 and reduced MMP activities (Figure 1). These data suggest LEN treatment suppressed VSMC phenotypic switching by promoting the RUNX1/SLC44A2 axis and subsequent activation of TGF-β/SMAD signaling (7).

## Concluding remarks and perspectives

Song and colleagues (7) found that *SLC44A2* is the key gene regulating VSMC phenotypic switching. The SLC44A2/NRP1/ITGB3TGF-β/SMAD signaling pathway is critical for maintaining VSMCs in their contractile state and disruption of this pathway triggers the phenotypic switch to a synthetic state, contributing to the development and progression of AA. The authors further showed that LEN treatment upregulated SLC44A2 expression and reduced AA development. These exciting findings provide a mechanism underlying the phenotypic switch of VSMCs and AA pathogenesis and the therapeutical potential of targeting SLC44A2 signaling for AA prevention and treatment. However, large cohort studies in human patients with AA are necessary to validate the efficacy of LEN-upregulated SLC44A2 in future clinical trials.

In conclusion, the study by Song et al. (7) identifies SLC44A2 as a regulator in VSMC phenotypic switching, providing evidence of its critical role in AA development. Their findings offer important insights into the mechanism underlying AA pathogenesis and suggest a pharmaceutical avenue for the prevention and therapy of AA.

## Version 1 08/15/2024

Electronic publication
